# P-1637. Four-year Experience on Ambulatory Antibiotic Prescribing for Viral Respiratory Infections

**DOI:** 10.1093/ofid/ofae631.1803

**Published:** 2025-01-29

**Authors:** Joanne Huang, Chloe Bryson-Cahn, Cameron Buck, Mike Hori, David Lin, Tanya Rizzo, Yilin Zhang, Zahra Kassamali-Escobar

**Affiliations:** Valley Medical Center, Renton, Washington; Harborview Medical Center, Seattle, Washington; Valley Medical Center, Department of Emergency Medicine, Renton, Washington; Valley Medical Center, Department of Infectious Diseases, Renton, Washington; Valley Medical Center, Renton, Washington; Valley Medical Center, Valley Family Medicine, Renton, Washington; Valley Medical Center, Valley Family Medicine, Renton, Washington; University of Washington Center for Stewardship in Medicine / Fred Hutchinson Cancer Center, Seattle, Washington

## Abstract

**Background:**

In 2019, our institution implemented the MITIGATE (a Multifaceted Intervention to Improve Prescribing for Acute Respiratory Infection for Adult and Children in Emergency Department and Urgent Care Settings) Toolkit, a multimodal antimicrobial stewardship strategy using education, data, and feedback to reduce unnecessary antibiotic prescribing for viral respiratory infections (RTIs) in ambulatory care settings.

**Methods:**

This was a single center observational study. Prescribers in emergency department (ED), primary care (PC), and urgent care (UC) were notified of their individual unnecessary antibiotic prescribing from 9/2019 - 3/2022 via email. We reduced rate of feedback from notifying all prescribers, to half after the first year, and none after 2 years and observed the impact on antibiotic prescribing. Prescribers were labeled as a top performer or not a top performer compared to their peers. Unnecessary antibiotic prescribing for RTI was evaluated 4 years after the intervention began.

**Results:**

In 2019 Pre-toolkit intervention, 14,247 patient visits had a qualifying viral RTI. 17.9%, 9.8%, and 6.4% received an antibiotic unnecessarily in the ED, PC, and UC, respectively. After 2-years of direct feedback, antibiotics were prescribed in 13.4%, 4.3% , and 4.7% , in the ED, PC, and UC (N = 48,192) (p< 0.001). In 2023, after feedback stopped, unnecessary prescribing remained lower in ED (14.4%, p< 0.001) and PC (5.8%, p< 0.001), but not UC (7.2%, p=0.015), N =53,496.

**Conclusion:**

Rates of unnecessary antibiotic prescribing for viral RTI dropped most significantly following two years of varying degrees of direct feedback. Although prescribing rates in the ED and PC remain lower after 4 years compared to pre-intervention, current data demonstrate an uptrend. Although labor-intensive, this supports that feedback with peer comparison is an instrumental intervention. Strategies to ensure successful intervention maintenance should be considered when implementing outpatient stewardship.

**Disclosures:**

**All Authors**: No reported disclosures

